# Increasing Consistency of Disease Biomarker Prediction Across Datasets

**DOI:** 10.1371/journal.pone.0091272

**Published:** 2014-04-16

**Authors:** Maria D. Chikina, Stuart C. Sealfon

**Affiliations:** 1 Department of Computational and Systems Biology, University of Pittsburgh, Pittsburgh, Pennsylvania, United States of America; 2 Department of Neurology, Center for Translational Systems Biology and Department of Neurology, Mount Sinai School of Medicine, New York, New York, United States of America; Yale University, United States of America

## Abstract

Microarray studies with human subjects often have limited sample sizes which hampers the ability to detect reliable biomarkers associated with disease and motivates the need to aggregate data across studies. However, human gene expression measurements may be influenced by many non-random factors such as genetics, sample preparations, and tissue heterogeneity. These factors can contribute to a lack of agreement among related studies, limiting the utility of their aggregation. We show that it is feasible to carry out an automatic correction of individual datasets to reduce the effect of such ‘latent variables’ (without prior knowledge of the variables) in such a way that datasets addressing the same condition show better agreement once each is corrected. We build our approach on the method of surrogate variable analysis but we demonstrate that the original algorithm is unsuitable for the analysis of human tissue samples that are mixtures of different cell types. We propose a modification to SVA that is crucial to obtaining the improvement in agreement that we observe. We develop our method on a compendium of multiple sclerosis data and verify it on an independent compendium of Parkinson's disease datasets. In both cases, we show that our method is able to improve agreement across varying study designs, platforms, and tissues. This approach has the potential for wide applicability to any field where lack of inter-study agreement has been a concern.

## Introduction

Microarray based expression profiling is widely used to investigate molecular changes associated with disease states and has the potential to elucidate clinically useful biomarkers that can be used for diagnosis, monitoring or personalized treatment. Since studies with human subjects often have limited size, meta-analysis methods that seek to improve the detection of differentially expressed genes through aggregation have received considerable attention. The varying approaches include explicit parametric models of error in gene expression measurements [1]–[4], heuristic aggregation of top differentially expressed genes [5],and using literature derived knowledge to find common network based patterns [6]–[8]. However, a key question that is typically not addressed in these methods is how to deal with studies that produce discordant results despite addressing similar biological questions. A lack of inter-study concordance is a common finding in human datasets [8]–[10], and it is well established that combining disagreeing or outlier studies can reduce the statistical power as well as lead to erroneous conclusions [11]–[14]. This becomes a problem of increasing concern as meta-analysis efforts are scaled up via searchable databases that allow biologists and clinicians to aggregate differentially expressed genes across related studies without carrying out explicit statistical analysis [15], [16].

Multiple sclerosis (MS) is a relatively common autoimmune disease that provides an informative case study in biomarker discovery and validation. Heterogeneity of clinical subtypes, episodes of relapse and remission, and varying response to treatment make the progression of the disease unpredictable and difficult to evaluate [17]. For that reason, there is considerable interest in the establishment of reliable molecular biomarkers that can be used to diagnose and monitor the disease. To improve our understanding of the molecular mechanisms involved, a number of microarray studies comparing various clinical groups have been undertaken. While many of the studies produce intriguing results on the nature of immune dysregulation [18]–[27], most findings have not been confirmed in independent studies, and MS biomarker discovery has been hampered by this lack of reproducibility. For example, the report that IL17F serum concentration was predictive of a lack of response to interferon therapy [28] was not confirmed by a subsequent study [29]. Meta-analytic approaches that combine data from different studies have the potential to elucidate biomarkers that are more likely to capture the underlying disease biology rather than differences limited to the specific patient cohort.

In this study, we demonstrate a novel statistically based approach to meta-analysis using a compendium of publicly available studies. Rather than simply aggregating the datasets for greater statistical power, we propose a method that improves inter-study agreement by applying an automated statistical correction to datasets on an individual basis. Our approach is based on the method of Surrogate Variable Analysis (SVA) [30] which uses the correlation structure of each dataset to estimate and correct for latent sources of variation. However, we demonstrate that the correlation structure of human disease studies may deviate considerably from the assumptions underlying SVA and propose an alternative approach that is essential to producing the improved agreement we observe. We demonstrate that the modified method improves agreement in our MS compendium and we verify this effect in an independent set of Parkinson's disease datasets. While it is often possible to extract more meaningful information from individual datasets by applying ad hoc analysis methods, we demonstrate that latent variable correction is a generalizable approach that can be applied in bulk to public datasets to achieve improved results.

## Results

The fundamental underlying assumption of MS biomarker studies is that there is a disease associated neuroinflammatory signature that can be observed in gene expression measurements. In principle, meta-analysis should be able to elucidate genes that may not be the top differentially expressed candidates in any one study, but that are nevertheless reproducibly changed across multiple expression profiles and are thus more likely to be related to the underlying disease process.

The current understanding of disease pathology is that it involves the infiltration of CNS tissue by blood derived leukocytes, and existing therapies target either immune cell activation or blood-brain permeability [31], suggesting that aberrant regulation of these processes should be detectable at the molecular level. In order to evaluate whether a common molecular signature can be observed in gene expression measurements of multiple sclerosis patients, we compiled a compendium of publicly available multiple sclerosis datasets (Table 1). While the complexity of this disease has led to studies of varying designs, all the studies in our compendium aim to evaluate patient groups with different multiple sclerosis phenotypes, and molecular changes that segregate these groups should in principle reflect the underlying neuroinflammatory process.

**Table 1:** Datasets included in our MS compendium.

Dataset	Platform	Factors	Tissue
E-MTAB-358	Illumina(HT-12 V3.0)	Disease[RRMS(12), PPMS(14), SPMS(16), Control(30)]	PBMC
E-MTAB-69	U133-Plus2	Disease[MSrelapse(12), MSremission(14), non-inflamatory(18)]	CSF, PBMC
GSE14895	U133A, U133A-2	Disease[CIS(13), Control(4)]	PBMC
GSE15245	U133A, U133A-2	Disease[CIS(18), MS(11)]	PBMC
GSE16461	U133-Plus2	Disease[MS(8), Control(8)], Cell[CD4,CD8]	T cells
GSE17048	Illumina(HT-12 V3.0)	Disease[RRMS(36), PPMS(43), SPMS(20), Control(45)]	Whole blood
GSE17449	U133A-2	Disease[MS(17), Control(11)], Pregnancy[Yes,No]	PBMC
GSE19285	U133-A,B	Prognosis[good(17), poor(7)]	PBMC
GSE23832	HuGene-1.0st	Disease[MS(8), control(4)]	PBMC
GSE24427	U133-A,B	Prognosis[good(18), poor(7)]	PBMC
GSE26484	U133-Plus2	Disease[MS(6),control(4)], Sem4A[low,high]	PBMC
GSE26927	Illumina(Ref-8 v2.0)	Disease[MS(10), other(10)]	Brain

### Multiple sclerosis studies have low agreement

In order to accommodate studies with multi-group designs, we employ analysis of variance for determining differential expression, a technique that has been previously used in a multi-group meta analysis setting [32]. We expect that the number of reproducibly altered genes to be small and in order to quantify the extent of non-random overlap in differential expression ranking within our compendium we follow the “concordance at the top” approach proposed in [33]. We select the top 5% of the genes in each dataset and score the overlap between all pairs of datasets using a hypergeometric test.

**Figure 1:**
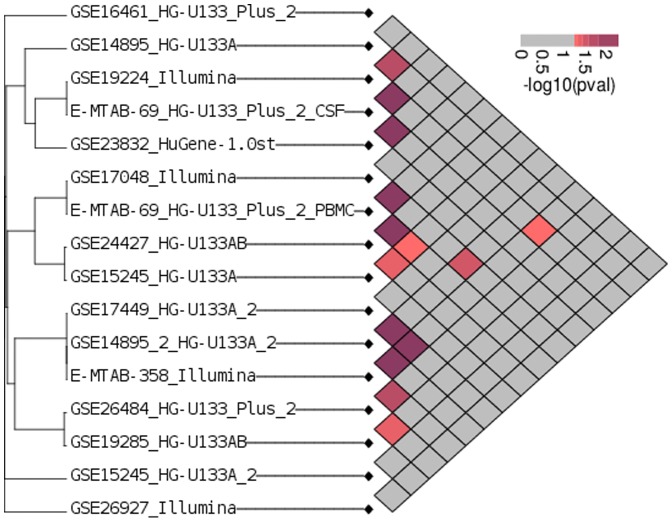
Quantifying agreement among multiple sclerosis datasets. Genes are ranked for differential expression by an F-test with respect to the multiple sclerosis phenotype. The degree of the overlap between lists of the top 5% of differentially expressed genes is evaluated for significance against a null distribution obtained by permuting the sample labels. The relative significance of the overlap study pairs is indicated by the color scale. The overall agreement among lists of genes predicted to be associated with multiple sclerosis phenotypes is low.

The hypergeometric test does not produce a true p-value in this case, as genes are not independent of one another, and not all genes have the same chance of being called differentially expressed. For that reason, we generate an empirical null distribution specific to each pair of datasets by permuting group labels in the two datasets and computing consensus tests from the resulting randomized data (see Methods for details). The resulting null distributions are then used to compute empirical p-values for the hypergeometric score. The results of this analysis are displayed in Figure 1.

We observe a general lack of consensus, as most datasets pairs have overlap no greater than what would be expected from randomized data. Given the diversity of our compendium, we expect that not all study designs should produce agreement, even in principle, and we expect outliers as well as groups of studies with similar designs that would manifest as clusters of high consensus. However, we do not observe such clusters. In fact, the overall distribution of empirical p-values is not significantly different from uniform (KS test p-value = 0.1215). The low overlap of multiple sclerosis microarray studies has been noted previously [34], and there are several possible explanations for this finding. There could in fact be no underlying biological signature that is common to the various patient cohorts and can be detected in the gene expression profile of immune cells. The other possibility is that the studies are simply too noisy relative to the sample size to detect any such signature.

Although a simple power calculation would dictate that a large sample size is required to detect what we expect are small transcriptional alterations in measurements with high variance, much of this variance may not be due to noisy measurements per se but may result from variation in genetic background, sample preparation, and other demographic and technical variables. Variation that is due to such variables can be largely corrected for by their explicit inclusion in downstream analysis. For that reason, most human studies will report demographic information such as gender and ethnicity. However, other biological variables, such as detailed genetic information, may be too costly to record and many of the technical variables are simply unknown; thus, the resulting variation cannot be modeled directly.

It was recently shown by Leek and Storey that, as such latent variables affect the expression of many genes at once, they can in principle be recovered from the dataset correlation structure via a procedure termed surrogate variable analysis (SVA) [30], [35]. This procedure produces “surrogate variables” that span the space covered by the latent variables, and can be treated similarly to any known covariate. Including the surrogate variables in differential expression analysis can dramatically alter the differential expression results and the authors demonstrate via a simulation study that applying SVA produces ranked gene lists that are more stable across repeated experiments. While SVA has been demonstrated to have favorable properties in a variety of real data applications, its rank-stabilizing effect has not been investigated in a practical setting. Given that the algorithm has the potential to improve reproducibility in theory, we hypothesized that it is indeed possible to use SVA as a normalization step when integrating public disease related studies where a common disease signature may be masked by dataset specific latent variables that conspire to produce discordant results.

We applied the SVA algorithm as implemented in the current Bioconductor version (SVA version 3.6.0) to our MS compendium. Although the ranked list of differentially expressed genes was significantly altered, no overall improvement in concordance was observed. In principle, this was not surprising as we expect to see improvement only in the cases where reproducible signal is masked by variance induced by latent variables. There is no guarantee that this condition is met in the MS compendium. However, upon closer inspection, it became apparent that for some datasets the SVA algorithm was producing surrogate variables that had significantly different means within the experimental groups. Including them in the analysis as covariates significantly reduced the differential expression signal, thus producing nearly random gene rankings.

### Heterogeneous mixture samples complicate latent variable estimation

The SVA algorithm is based on the observation that if a gene-by-sample matrix *X* arising from a microarray experiment is modeled as
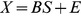
(1)where *S* is the study design matrix (which includes the primary effect such as disease status as well as any known covariates), the rows of the residual matrix *E* are not independent random errors as is typically assumed, but are correlated. In fact, the data is better modeled as
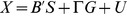
(2)where *G* represents the latent variables that explain the residual correlation of *E*, and the rows of *U* are truly independent. The SVA algorithm is aimed at estimating *G* by exploiting dataset correlation structure.

A simple procedure to estimate the latent variable space would be to perform singular value decomposition (SVD) on the residual matrix, 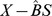, however this assumes that any latent variables are completely balanced among the experimental groups, which is not true in practice. In order to allow latent variables that are non-orthogonal to the main effect, the algorithm of Leek and Storey employs a complex iterative procedure, whereby the singular vectors from the residual SVD are used as initial estimates of *G*, and the surrogate variables are recomputed using a reweighting of the genes in the original data matrix.

The pseudocode for the iterative procedure is as follows:

**Require:** Gene expression matrix *X*, design matrix *S*

1: Estimate *k*, the dimensionality of the latent variable space {Methods to do this are described in detail elsewhere [30], [35]}

2: Fit model *X* = *BS*+*E*

3: Perform singular value decomposition (SVD) of the residual 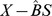


4: Set G to the first *k* eigenvectors {This initial estimate of *G* is orthogonal to *S*}

5: **for** i in 1:B{number of iterations} **do**

6:   Compute gene weights using the posterior probability that a gene has an association with *G* but has no association with *S*

7:   Perform SVD of a weighted matrix

8:   Set G to the first *k* eigenvectors

9: **end for**

10: **return** *G*

For many studies within our MS compendium this procedure produced one or two surrogate variables that were heterogeneous, i.e. showed highly significant differences between groups. While it is possible that the studies are not well randomized and some technical variable is correlated with the phenotype of interest in all of these cases, a detailed investigation revealed a surprising pattern inconsistent with this conclusion.

**Figure 2:**
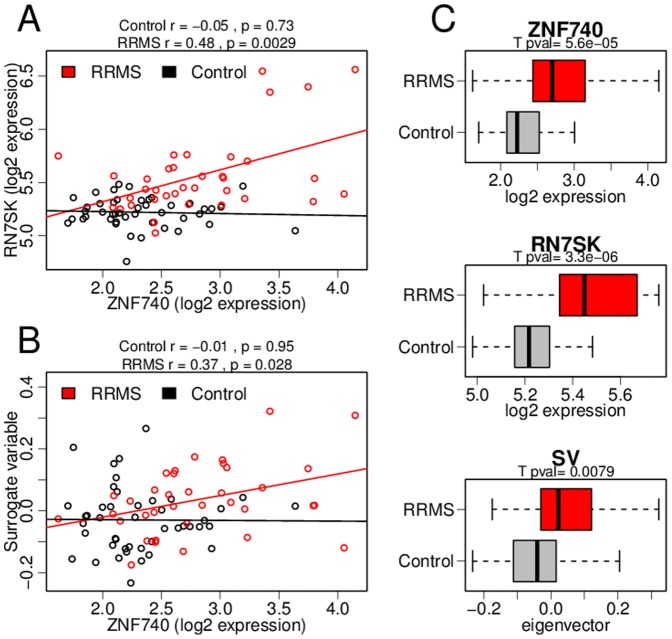
Disease-specific correlation produces heterogeneous surrogate variables. When SVA is applied to the multiple sclerosis dataset GSE17048, some of the resulting surrogate variables are unequally distributed among the experimental groups. These surrogate variables (SV) follow the pattern of disease specific correlation present in this dataset. (A) An example of a correlated pair of top ranked differentially expressed genes. The group specific correlation coefficients and their significance is noted above the plot: the correlation between these genes is only observed in the relapse-remitting MS (RRMS) samples. (B) The disease specific pattern of correlation is captured in a surrogate variable. (C) The surrogate variable in (B) recapitulates the differential expression observed in the individual genes.

The heterogeneous surrogate variables were most pronounced in the largest and most comprehensive study in our compendium, GSE17048. Indeed, differentially expressed genes could not be deconvolved from the latent structure of the dataset; they tended to be correlated with each other. However, the pattern of correlation was different from what might be expected if it were driven by confounding variables. For many differentially expressed genes the correlation was only observed in the disease state (Figure 2 A for an illustrative example of group specific correlation).

Rather than representing non-randomized latent variables we argue that this effect arises from a more fundamental phenomenon that is particular to complex tissue samples. Tissue samples obtained from human subjects tend to vary considerably in their cell-type composition and this is especially true of human blood where the proportion of different cell-types can vary four fold in healthy individuals [36]. Moreover, when assaying mixture samples we expect that for some genes the expression is altered in a cell-type specific manner. We believe that this effect in conjunction with the overall cell type composition variation is responsible for the unusual correlation structure which in turn makes SVA difficult to apply.

We model this situation in order to study its impact of differential expression analysis. For simplicity let us assume that there are three different cell types and the observed differential expression is arising from an altered state in one of the cell types. Formally, if *e_i_*_,*j*_ denotes the expression of a gene *i* in cell type *j* and *p_j_*_,*k*_ denotes the proportion of cell type *j* in sample *k* the expression measurements can be modeled as
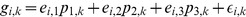
(3)for the control group and as
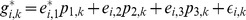
(4)for the disease group, where 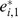 represents a disease related expression state in cell type 1.

If we consider a set of genes that in the control population are expressed at approximately the same level in all the cell types in the mixture so that 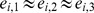, then in the control population the expression of these genes will be independent of sample-specific cell type compositions and the series of measurements 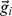 can be modeled as
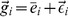
(5)where 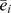 is the average expression value across the three cell types and 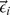 is random error. Consequently, genes in this category will not show correlation with each other.

If however, in the disease state, these genes are overexpressed in a particular cell type, so that we have 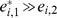 and 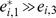 their expression values will be dominated by the expression in that single cell type, so that the measurement can be approximated as
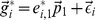
(6)These expression measurements will correlate with the proportion of the first cell type in each sample, 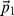, thereby making the genes correlate with each other.

**Figure 3:**
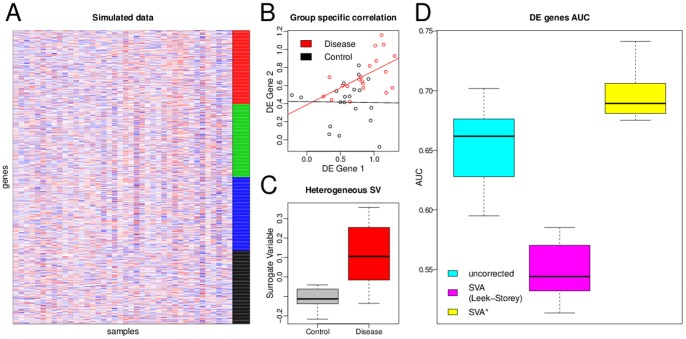
Performance of different SVA methods in simulated mixture dataset. We simulated a microarray dataset derived from a heterogeneous mixture of 3 different cell types. The 1000 simulated genes were assigned 4 classes: 250 are background noise (black), 250 have a group effect (red), 250 are affected by an artificial latent variable (blue), and 250 are affected by both (green). The group effect is modeled as differential expression in one of the three cell types. (A) Heatmap of an example dataset with gene class denoted in the color on the right. Aside from the structure imposed by the group effect and the linear latent variable both affecting 500 genes the dataset has global correlation structure, imposed by the mixture model. (B) Under this model we are able to recapitulate the group specific correlation in the differentially expressed genes that we observe in real datasets, see example in Figure 2. (C) Applying SVA to the simulated datasets produces surrogate variables that are strongly correlated with the experimental group. (D) Boxplot representing distribution of AUCs, area under receiver operating characteristic curve, for discriminating the green differentially expressed genes from the blue and black sets resulting from 20 repeats of the simulation. While the original SVA algorithm does not perform well in this simulation, the modified algorithm is robust to the confounding latent structure.

In order to demonstrate how this data structure effects differential expression analysis, we follow the procedure similar to that outlined in [30] to generate a simulated dataset. We generate a dataset with two groups and a single artificial latent variable. Of the 1000 simulated genes, 250 are background noise, 250 have a group effect, 250 are affected by the latent variable, and 250 are affected by both. However, unlike the procedure in [30] the baseline expression is modeled not as random values but as a random mixture of the expression vectors of 3 different cell types with differential expression arising from an altered expression state in one of the three cell types (as in equations 3 and 4). Under this model, we are able to reproduce the group-specific correlation in the differentially expressed genes (Figure 3B) which in turn leads the SVA algorithm to estimate a heterogeneous latent variable (Figure 3C).

In order to remedy this problem, we propose a modification of the Leek-Storey SVA algorithm which uses a different function to weight the genes (step 5 in the pseudocode above). The goal of SVA and related methods can be viewed as partitioning the variation in the dataset into that which is due to legitimate (and thus reproducible) difference between the clinical groups and that which is due to latent variables (which may be confounded with the group effect). This is accomplished by computing SVD on a reweighted matrix and the original approach is to use local FDR as the posterior probability that a gene is affected by latent variables, *G*, and is not affected by the primary group effect. While this approach is statistically grounded, it does not work well on mixture datasets, as the posterior probabilities eventually converge to values near 1 thus weighting most genes similarly. In this case surrogate vectors approximate the eigenvectors of the unweighted SVD and thus capture all the variation in the dataset, including the differential expression. While this is desirable in cases when the groups are not randomized with respect to some technical or biological variable, this becomes problematic in complex mixture samples where differential expression may be confounded with the correlation structure even under perfect randomization. Though variations of the original SVA method have been proposed, most notably SVA-PLS [37] and ISVA [38], they do not address this aspect of latent variable estimation and perform no better on the simulated mixture dataset (see Figure S1)

We propose an alternative weighing approach that is less aggressive. In place of the posterior probability our approach uses the raw p-value for both the primary and the latent effects. In doing so we are not equating the p-values with posterior probability but rather using them as an alternative gene-weighing scheme which has the effect of restricting the degree to which surrogate vectors can vary among the clinical groups. We compute the p-value via a permutation test, since in real datasets we observe genes that do not conform to a normal distribution. Using the p-value directly has the effect of generating surrogate variables that only show association with the primary effect that might be expected by chance alone in a completely randomized study. The resulting surrogate vectors are thus still allowed to be non-orthogonal and may in fact show large deviations from orthogonality if the sample size is small however this approach prevents the generation of surrogate variables with significantly different group means. While this method will not fully recover the correct latent structure when studies are not well randomized, it is able to handle correlation that is induced by interaction with the primary variable, as is the case in mixture samples.

To illustrate the effect of our approach, we apply the different methods to the simulation described above. SVA should in principle increase our power to detect the 250 differentially expressed genes that are also affected by the artificial latent variable. Because in this simulation the correlation structure of the data is complex, and includes correlation induced by the mixture model itself, the SVA algorithm produces multiple surrogate variables some of which have significantly different within group means and the intended effect of SVA is canceled out. Our modification, however, is robust to this effect and, as intended, is able to improve the detection of differentially expressed genes (Figure 3D).

### Modified SVA* algorithm successfully improves inter-study agreement

**Figure 4:**
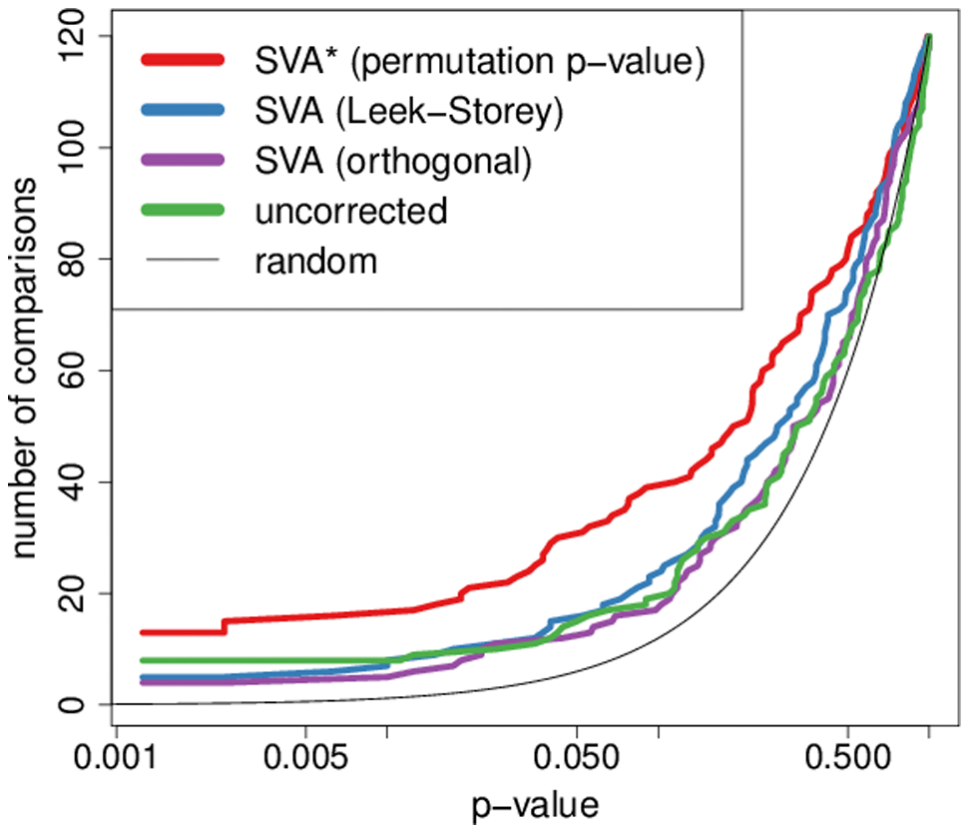
Comparisons of the inter-study agreement generated by various SVA algorithms. For each p-value threshold we plot the number of pairwise comparisons (out of 120 possible) reaching that level of significance. SVA* produces the most comparisons with small p-values. The overall differences between the agreement curves plotted were evaluated using a single tailed signed rank test on the log transformed empirical p-values which evaluates the hypothesis that SVA* improves the significance of individual pairwise comparisons. Overall agreement using the modified SVA algorithm was significantly improved over the uncorrected analysis (p = 0.015) and the Leek-Storey SVA (p = 0.022).

Using the modified SVA* algorithm we were able to achieve a significant improvement in dataset concordance within our MS compendium, confirming our initial hypothesis that latent variable modeling can be effective in a meta-analysis context. The method is applied to each dataset independently, and it doubles the number of significant pairwise overlaps between sets of differentially expressed genes over what is produced by standard differential expression analysis (Figure 4). The modified SVA* algorithm also produces much better agreement than the original SVA algorithm, demonstrating that our modification to the weighting function is crucial to achieving the desired effect.

Since we demonstrate that the weighting function and heavily biased surrogate vectors are indeed the problem, it is natural to investigate how the algorithm would perform with no weighting at all. Leek and Storey reject this approach of using the initial orthogonal surrogate variable estimates. While they believe it would be effective at correcting for latent structure, it produces anti-conservative p-values. In our study we are not concerned with p-values, but rather with gene ranking; nevertheless, we find that the orthogonal method is not effective at improving dataset concordance, demonstrating that some variation of the weighting approach, which allows surrogate variables to correlate with clinical variables, is required in order to observe improvement in agreement.

**Figure 5:**
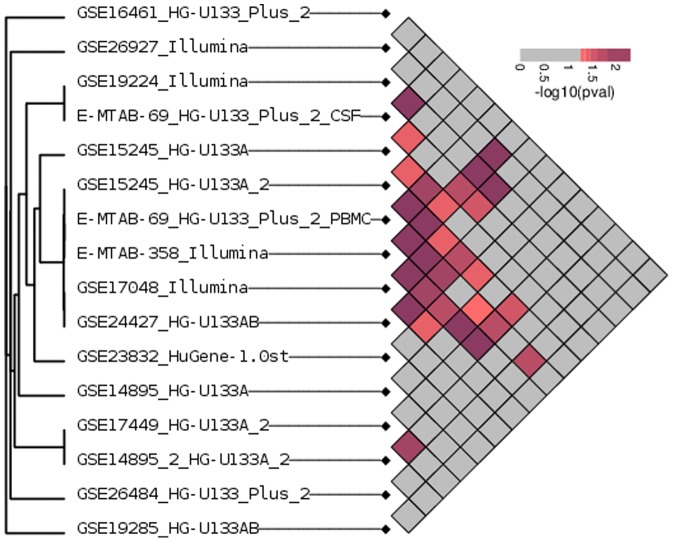
Heatmap of inter-study agreement after modified SVA correction. We observe dramatic agreement over a simple F-test using explicitly provided covariates only (uncorrected) as well as the unmodified SVA.

We have demonstrated that our modified SVA approach is effective at improving overall dataset agreement. However, the particular pattern of agreement (Figure 5) gives us further confidence that this improvement is biologically meaningful. While the naive approach produces agreement that barely deviates from what is expected by chance, with pairs of agreeing datasets seemingly randomly distributed, the modified SVA approach reveals a cluster of highly overlapping studies. Importantly, several of the studies that cluster together are studies that are *a priori* expected to be of high quality because they have large sample sizes. The clustering is also independent of platform, which further supports its biological relevance.

### The modified SVA* algorithm also improves inter study agreement in an independent compendium of Parkinson's disease datasets

While our modified SVA algorithm was developed from basic considerations and we did not perform any parameter fitting or optimizations, our approach was informed by a detailed investigation of the datasets in our MS compendium. In order to confirm that the proposed SVA* algorithm can be useful in multiple contexts we use the algorithm without modification on an independent gene expression compendium of PD datasets. This compendium comprises publicly available datasets used in a recent Parkinson's meta-analysis [39], and differs significantly from our MS compendium. Most of the datasets in the Parkinson's compendium use brain samples, with many from the regions directly affected by neuron loss (Table 2), and are thus studying the tissue where macroscopic disease pathology can be observed. Thus, we expect that the disease signal should be considerably larger which in turn should lead to better inter-dataset correspondence. Indeed, we find that unlike in the MS compendium, the baseline uncorrected correspondence is appreciably better than random.

**Table 2:** Datasets included in our Parkinson's compendium.

Dataset	Platfrom	Tissue	Samples(PD/controls)
GSE22491	Agilent(G4112F)	PBMCs	8/10
GSE20159	Illumina(HT-12 V3.0)	Brain, substantia nigra	16/17
GSE20141	U133-Plus2	Brain, substantia nigra	8/10
GSE20146	U133-Plus2	Brain, globus pallidus interna	8/10
GSE20163	U133A	Brain, substantia nigra	10/10
GSE20164	U133A	Brain, substantia nigra	9/8
GSE20168	U133A	Brain, prefrontal cortex	15/14
GSE20291	U133A	Brain, putamen	20/15
GSE20292	U133A	Brain, substantia nigra	18/11
GSE20314	U133A	Brain, cerebellum	4/4
GSE8397	U133A	Brain, substantia nigra	18/29
GSE7307	U133-Plus2	Brain (multiple)	182/26
GSE7621	U133-Plus2	Brain, substantia nigra	9/16
GSE6613	U133A	Whole blood	22/50

We expect that the agreement can be improved further with an application of latent variable correction. However, like blood, brain tissue is a complex mixture of multiple cell types and heterogeneity in relative cell proportions may be exaggerated in the disease state [39], [40]. Hence, brain samples can produce the same kind of confounded correlation structure that interferes with latent variable correction making our modified SVA algorithm particularly useful.

**Figure 6:**
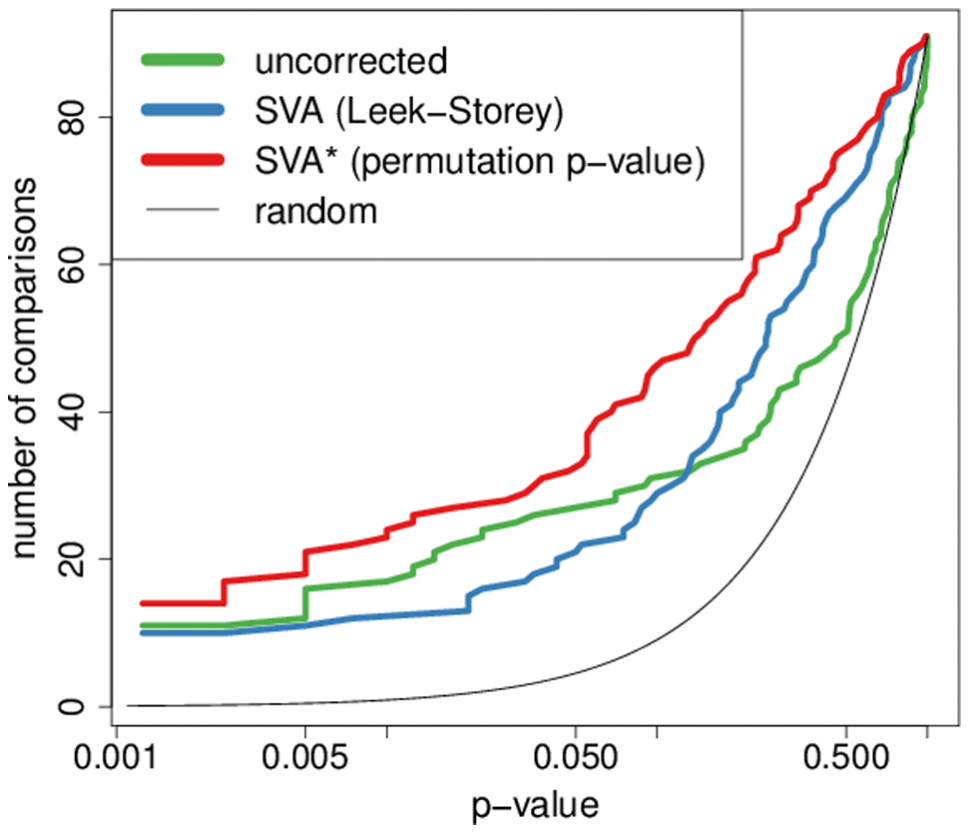
Improvement in inter-study agreement in the Parkinson's compendium. Comparisons of the inter-study agreement generated by various SVA algorithms. For each p-value threshold we plot the number of pairwise comparisons (out of 91 possible) reaching that level of significance. The overall differences between the agreement curves plotted were evaluated using a single tailed signed rank test on the log transformed empirical p-values. Overall agreement using the modified SVA algorithm was significantly improved over the uncorrected analysis (p = 6.8·10^−6^) and the Leek-Storey SVA (p = 1.8·10^−4^).

As is the case with the MS compendium, no improvement is observed with the original, Leek-Storey, SVA algorithm. However, confirming our expectations, the modified SVA* algorithm is able to significantly improve the inter-dataset concordance producing a greater number of comparisons with small p-values (see Figure 6).

Unlike multiple sclerosis PD is a disease for which concrete molecular mechanisms are established, making it possible to evaluate the results from a biological rather than statistical point of view. For example, SNCA, a gene whose product is known to be directly involved in disease pathology is downregulated in many of the datasets (ranked in the top 10% for 7 out of the 14 datasets) and applying SVA* improves its overall ranking (SNCA is 5th instead of 11th in mean rank). One interesting observation is that SVA* is particularly effective at improving the agreement of a whole blood dataset GSE6316 (which we expect is subject to large cell proportion variation) with datasets that assayed brain tissue. Changes in the expression of SNCA can be observed in this blood dataset even without any correction but applying SVA* shifts SNCA differential expression ranking from 139 to 34 (out of 9062 evaluated genes). On the other hand using Leek-Storey SVA SNCA is ranked 3312, further corroborating our hypothesis that the original SVA algorithms is most compromised in mixture datasets with large proportion variation.

## Discussion

Given that reproducibility in microarray studies with human subjects is a recognized problem, alternative analysis methods that are capable of resolving disagreements are of great interest. Improved concordance would provide independent validation for findings of individual studies, improve our ability to do meta-analysis, and provide more trustworthy predictions regarding differentially expressed genes. Moreover, inter-study concordance presents a unique evaluation for the analysis methods themselves. Improved agreement, when carefully evaluated against permuted datasets, must be achieved by extracting more biologically meaningful information from the data. For example, it has been shown that pathway level analysis, which can improve power of individual studies, also improves the agreement among independent datasets [8].

We present a method that is able to achieve improvement in agreement through estimating and modeling latent variables. Our method builds on surrogate variable analysis, a previously described approach for latent variable estimation. However, we demonstrate that unique correlation structure present in complex mixture samples can compromise the effectiveness of SVA, and we propose a robust alternative that overcomes this problem. The fact that improvement in agreement could only be observed with the modified SVA* algorithm highlights the importance of understanding the correlation structure in detail. Further work is needed to determine whether additional improvements in SVA can be achieved, and whether other modifications will be necessary for different types of datasets. Currently SVA operates without any knowledge of gene identities and as such differentiating between confounded latent variables and correlation structure that is due to a legitimate biological phenomenon may not always be possible. It is likely that approaches that use control genes, such as one described in reference [41], can be combined with unsupervised SVD based techniques to achieve superior results.

Despite the potential for further improvements, our study has demonstrated that latent variable modeling can be used as an effective out-of-the-box pre-processing approach in integrating human disease datasets and thus can be applied effectively to extract valuable new insight from the huge number of existing datasets.

## Methods

### Data Processing

Expression data and associated information was downloaded from GEO or ArrayExpress. Affymetrix data was processed using GCRMA. Illumina data was background adjusted with the bg.adjust() function from the “affy” BioConductor package, log-transformed, and quantile normalized. Data for the Agilent dataset, GSE22491, was downloaded from GEO in the processed format supplied by the authors. Probes were mapped to gene names using GEO GPL files and when several probes mapped to the same gene, the one with the maximum mean expression was kept as the representative. The number of genes before filtering varied from 13000 to 20000 depending on platform. The bottom 20% of genes with low expression or low variance were removed from each dataset before processing for differential expression. Using these criteria approximately 25% of the genes are filtered out since low expression and low variance genes largely overlap. Differential expression was evaluated with an F-statistic comparing a model that includes the disease related phenotype with one that includes only covariates. The covariates included gender and other experimental covariates provided by the authors (see Table 1). Corrected differential expression lists were produced by appending surrogate variables computed with various modifications of SVA to the design matrices.

### Overlap Computation

Our goal for the overlap computation was to evaluate consistency of gene rankings while taking into account that the number of differentially expressed genes is small and therefore ranking in the bottom of the list is not biologically meaningful. Thus, we evaluate the extent of nonrandom overlap in the top 5% of differentially expressed genes from each dataset. We compute raw pairwise overlap values as the hypergeometric probability *p*(*k*, *m*, *n*, *N*) where *m* and *n* are the number of genes in the candidate list of each dataset that was also present in the other dataset, *k* is the number of genes present in the overlap, and *N* is the total number of genes present in both datasets after filtering.

Null distributions of the raw overlap score were generated by permuting the phenotype labels of each dataset (while keeping covariates the same) and applying the differential expression pipelines to the permuted labels. For each dataset we generated 20 permutation based gene rankings and these were used to generate 400 dataset pair specific null overlap scores. Each dataset pair generates a unique null distribution with some being close to uniform while others are skewed towards small p-values due to gene-wise dependence. By comparing the real hypogeometric value to this distribution we arrive at an empirical p-value that corresponds to the amount of agreement we might expect by chance alone. We include a R script that applies this analysis to simulated mixture datasets in the supplement (File S1).

### Simulations

Our simulation is based on that described in Leek and Storey [30] with the alteration that the baseline expression of each gene (the expression unaffected by the latent variable or the group effect) is modeled not as independent random values but as a random mixture of 3 expression vectors representing pure cell types. The vectors are added in linear expression space and the data is subsequently logged. The dataset is divided in half to represent two experimental groups. Overexpression in the “disease” group is simulated by altering 500 genes in one of the pure cell expression vectors. We also model a artificial, that is distinct from those induced by the mixture model, latent variable as a random vector drawn from a uniform distribution. The gene specific effects for the latent variable are then drawn from a normal distributions and the outer products of the effect vector with the latent variable is added to the expression matrix. Finally, random noise is added to create the final matrix. The dataset was evaluated for differential expression using a T-test with no covariates and a T-test with covariates generated by the original SVA algorithm and our modified version. All the code necessary to run the simulation and reproduce Figure 3 is provided in the supplement (File S1).

## Supporting Information

Figure S1 **Performance of original Leek-Storey SVA and alternatives on a mixture dataset with cell type specific regulation** Simulation in Figure 3 was repeated with other SVA alternatives. Neither SVA-PLS or ISVA address the complex correlation structure of mixture datasets and do not improve differential expression discovery.
(TIF)

File S1 **Source code for modified SVA and simulations.**
(ZIP)
