# Supplementary figures and images for: Increasing Consistency of Disease Biomarker Prediction Across Datasets
Source: PLoS One. 2014 Apr 16;9(4):e91272. doi: 10.1371/journal.pone.0091272 (PMC3989170; doi:10.1371/journal.pone.0091272)

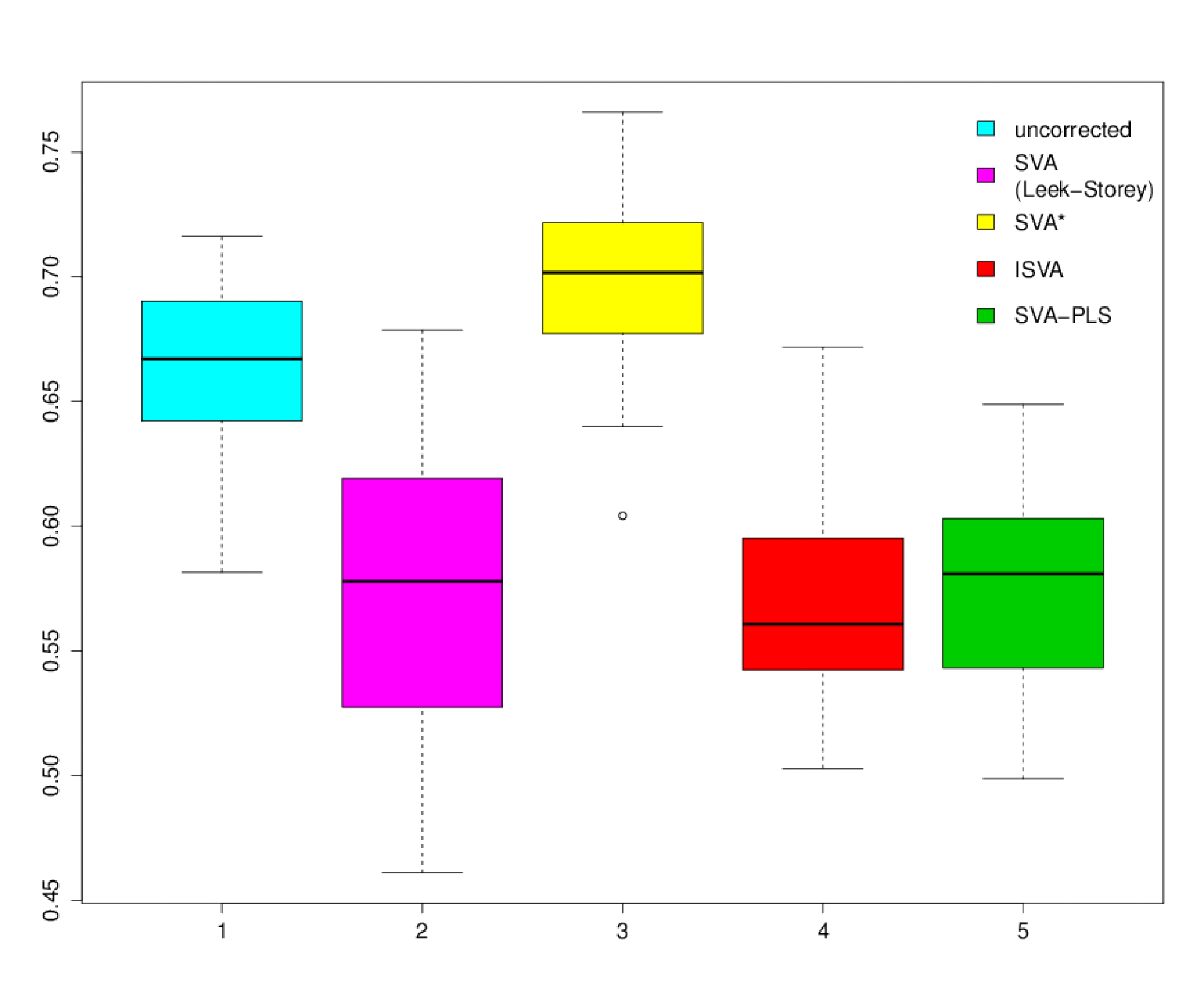

Supplement: Figure S1 — Performance of original Leek-Storey SVA and alternatives on a mixture dataset with cell type specific regulation Simulation in Figure 3 was repeated with other SVA alternatives. Neither SVA-PLS or ISVA address the complex correlation structure of mixture datasets and do not improve differential expression discovery. (TIF) [file pone.0091272.s001.tif]
